# Metazoan endoparasite fauna and feeding ecology of commercial fishes from Java, Indonesia

**DOI:** 10.1007/s00436-021-07377-4

**Published:** 2022-01-07

**Authors:** Svenja Koepper, Sri Nuryati, Harry W. Palm, Christian Wild, Irfan Yulianto, Sonja Kleinertz

**Affiliations:** 1 Marine Ecology Department, Faculty of Biology and Chemistry, University of Bremen, Leobener Str. 6, 28359 Bremen, Germany; 2 Aquaculture and Sea-Ranching, Faculty of Agriculture and Environmental Sciences, University of Rostock, Justus-von-Liebig-Weg 6, 18059 Rostock, Germany; 3 Faculty of Fisheries and Marine Sciences, IPB University; Adjunct Professor and DAAD Long-term Lectureship Fellow (SK), Department of Aquaculture (SN) and Department of Fisheries Resources Utilization (IY), Jl. Agatis Kampus IPB Dramaga, Bogor, Indonesia

**Keywords:** Parasite diversity, Stomach content analysis, Molecular analysis, Fish health, Seafood health risks, *Anisakis*, *Stephanostomum* cf. *uku*

## Abstract

Despite being an important component of the marine ecosystem and posing health risks to human seafood consumers, fish parasites in Indonesia have yet to be adequately described. Here, we analyzed the diet and metazoan parasite fauna of seven commercial fish species (*Alectis indica*, *Carangoides chrysophrys*, *Johnius borneensis*, *Mene maculata*, *Trichiurus lepturus*, *Upeneus asymmetricus*, *U. moluccensis*) landed in Java, Indonesia. We isolated 11 endoparasite species, established 22 new host and 14 new locality records, and extended parasitological records of *A. indica* by 24%, *C. chrysophrys* by 25%, *J. borneensis* by 40%, *M. maculata* by 44%, *U. asymmetricus* by 100%, and *U. moluccensis* by 17%. We genetically identified the trematode *Stephanostomum* cf. *uku* (of Bray et al. 2005) from *Alecta indica* for the first time in Indonesia and provided the sequence of its 28S marker. Stomach content analysis revealed seven different prey items, and the examined fish species were grouped into four feeding categories, which differed significantly in their respective endoparasite fauna. All but two examined fish species hosted potentially zoonotic nematodes, which reveal a risk for parasite-borne diseases in Indonesian food fishes and call for more consequent monitoring with regard to seafood safety in this region. With this study, we were able to establish an association between the feeding ecology and the endoparasite fauna of marine fishes which will help to better understand the transmission pathways of (potentially zoonotic) parasites in food fishes in tropical waters.

## Introduction

Endoparasites have a diverse range of effects on their hosts. In fishes, they can impair host growth, survival, reproduction, and mortality, transmit diseases, and affect the marketability of aquaculture and fisheries products (Farrell et al. 1964; van Banning and Haenen 1990; Yuasa et al. 2007; Barson 2008; Bouwmeester et al. 2020). Endoparasites, mainly anisakid and raphidascarid nematode larvae, can cause diseases in humans when accidentally ingested along with raw or undercooked fish. Known zoonotic genera are *Anisakis* spp., *Contracaecum* spp., *Hysterothylacium* spp., and *Terranova* spp. (Shamsi et al. 2018; Rahmati et al. 2020). For example, anisakiasis is a disease caused by third-stage *Anisakis* spp. larvae, and symptoms can range from intestinal pain to allergic reactions (Sakanari and McKerrow 1989; Slifko et al. 2000; Klimpel and Palm 2011; Mattiucci et al. 2018). Many zoonotic nematode species are found in tropical waters; for example, Palm et al. (2017) reported *A. berlandi* and *A. pegreffii* from *Auxis rochei* for the first time from Bali, Indonesia. Despite the implications for seafood health, the distribution patterns, transmissions, and epidemiology of zoonotic parasites in Indonesia remain largely unknown. As host migrations and marine food webs may change due to climate change (Palm et al. 2011; Worm and Lotze 2021), this research area should be a focus of the investigation.

Their complex life cycles involving multiple intermediate hosts and their sensibility to specific environmental conditions allow many endoparasites to be used as environmental indicators (Palm 2011; Kleinertz et al. 2016; Neubert et al. 2016; Vidal-Martínez et al. 2019). Marine parasitology is still a globally underrepresented field in aquatic biodiversity and ecological research, although it is an essential tool in aquatic health studies and to get insights into fish diet, fish stocks, migration, and trophic positions (Marcogliese and Scholz 1999; Marcogliese 2003; Kleinertz et al. 2012).

Stomach content analyses can reveal important information on the trophic interactions and the feeding ecology of fishes (Cox et al. 2002; Graham et al. 2007). Endoparasites are usually transmitted along the food web when intermediate, transport, or sometimes accidental fish hosts feed on parasitized prey (Poulin and Valtonen 2002). It is assumed that the diet of the fish host strongly influences its parasite abundance and richness (Cirtwill et al. 2015). Including host diet data in endoparasite studies can put a new perspective on parasite transmissions and infection patterns and give additional insights into the host’s ecology (e.g., Klimpel et al. 2006; Kleinertz et al. 2012).

Indonesia is the world’s largest island state (Harris 2001), and its population and economy largely depend on fishery and aquaculture (Tran et al. 2017). The fishing industry has been growing over the past decades, securing income and employment for the public (Jermsittiparsert et al. 2019). Now, Indonesia is the second biggest seafood producer and exporter after China (Hakimah et al. 2019; FAO 2020), demanding control mechanisms and ongoing studies concerning the reliable food safety of fisheries products. Located in the Coral Triangle, Indonesia’s high biodiversity is also reflected in the associated parasite fauna as parasite distribution is linked directly to the presence of their hosts (Allen et al. 2003; Palm and Rueckert 2009). In recent years, research on parasites of commercial fishes increased, but it is estimated that approximately 95% of species are yet unknown from Indonesian waters (Jakob and Palm 2006; Theisen 2020). Closing this knowledge gap is important to understand parasite-related health risks and to secure seafood safety, especially in climate-changing times, where species distributions are likely going to shift, potentially leading to altered zoonotic parasite loads in food fishes (Marques et al. 2010; Klimpel and Palm 2011; Ullah et al. 2018).

The present study identified the endoparasite fauna and the stomach contents of seven commercially important fish species from West Java (*Alectis indica* (Indian threadfish), *Carangoides chrysophrys* (longnose trevally), *Johnius borneensis* (sharpnose hammer croaker), *Mene maculata* (moonfish), *Trichiurus lepturus* (largehead hairtail), *Upeneus asymmetricus* (asymmetrical goatfish), and *U. moluccensis* (goldband goatfish))*.* This study aims to analyze their endoparasite community that, depending on the trophic level of each species, should differ. For that, we categorized the fish species based on their examined diet and compared the endoparasites community among these feeding categories: mainly fish feeder as a top predator, mainly fish feeder as smaller sized or schooling fish, mainly fish and decapod feeder, and mainly cephalopod feeder. Furthermore, we discuss the importance of the identified prey items as parasite transmitters and potential health risks to consumers through the consumption of the above seven species.

## Materials and methods

### Sample collection

**Fig. 1:**
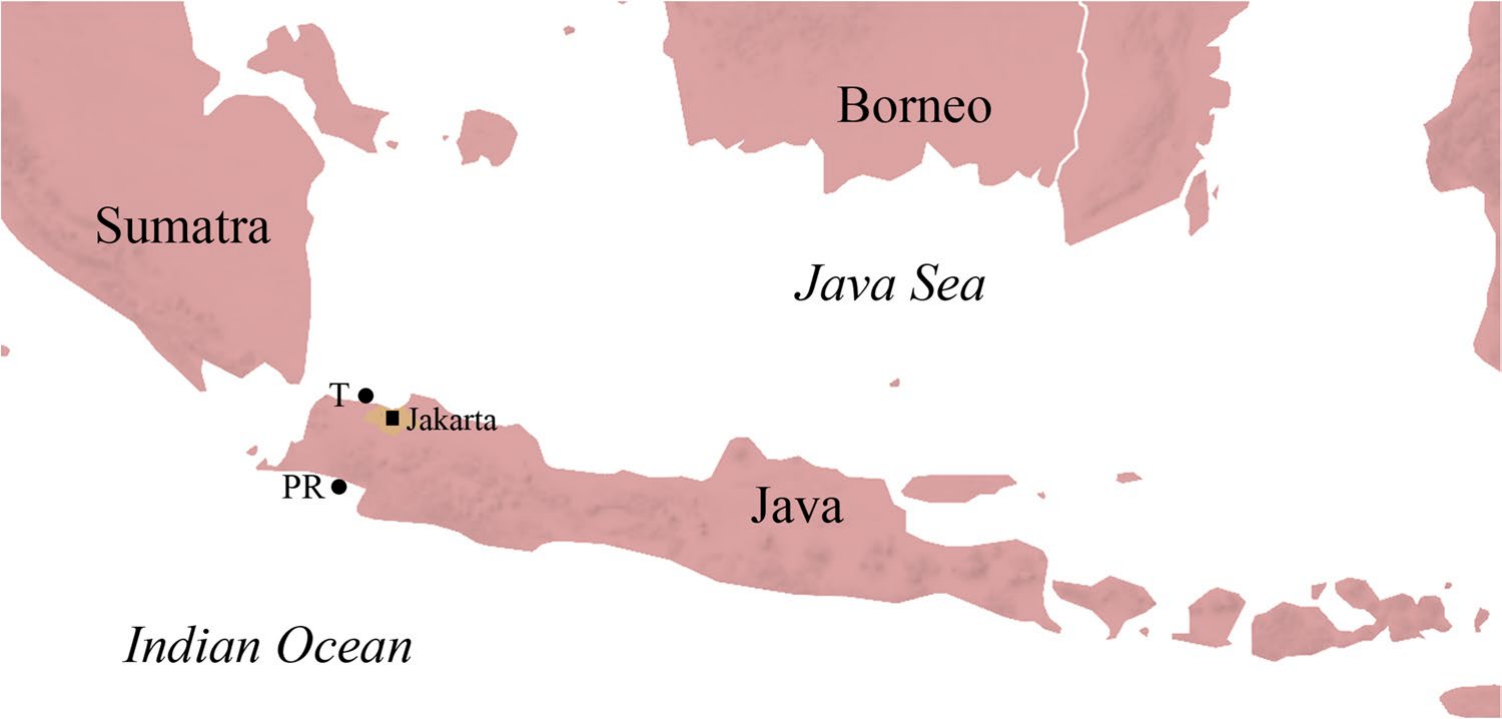
Map of study area on Java, Indonesia. Samples were collected from fish markets in Pelabuan Ratu (PR) and Tangerang (T). Map according to Koepper et al. (2021)

A total of 140 fishes of seven species (20 per species, a sufficient sampling size to estimate prevalence according to Jovani and Tella (2006)) were collected from April to June 2018 from fish markets in Pelabuhan Ratu and Tangerang in West Java, Indonesia (Fig. 1, Table 1). After purchase, the samples were deep-frozen in single plastic bags, usually for 2 to 5 days, until the day of analysis when they were defrosted in lukewarm water. Any fluids within the plastic bag were later examined under a stereomicroscope (Zeiss, Stemi DV4). Morphometric data of each fish was taken (total length, standard length, total weight, and slaughter weight) according to Palm and Bray (2014).

**Table 1:** Fish biological data of examined fish species, 140 specimens sampled in April 2018, with the sampling locations in brackets (PR, Pelabuhan Ratu; T, Tangerang)

Fish species	TL (cm)	SL (cm)	TW (g)	SW (g)	f	m	n.I.
*Alectis indica* (T)	71.1 ± 7.8	61.2 ± 7.5	2886.1 ± 807.5	2745.1 ± 744.6	1	18	1
	(54.3–82.3)	(45.3–71.2)	(1288.5–3969.3)	(1231.0–3830.7)			
*Carangoides chrysophrys* (T)	48.1 ± 2.7	41.9 ± 2.4	1477.4 ± 196.0	1349.2 ± 175.6	8	12	-
	(43.4–52.6)	(37.0–46.7)	(1185.4–1750.5)	(1103.0–1605.2)			
*Johnius borneenis* (PR)	17.2 ± 1.3	14.8 ± 1.1	75.4 ± 17.6	68.0 ± 15.2	9	11	-
	(15.2–19.1)	(13.3–16.6)	(48.8–105.6)	(46.6–93.1)			
*Mene maculata* (PR)	17.7 ± 1.1	14.8 ± 0.8	107.1 ± 15.5	99.1 ± 13.4	12	4	4
	(15.0–19.3)	(13.7–16.5)	(80.4–138.9)	(69.0–128.5)			
*Trichiurus lepturus* (T)	63.8 ± 4.1	63.8 ± 4.1^a^	250.4 ± 63.7	235.5 ± 55.5	5	15	-
	(55.9–69.3)	(55.9–69.3)	(141.4–415.7)	(134.3–379.3)			
*Upeneus asymmetricus* (PR)	19.2 ± 1.7	15.9 ± 1.5	88.3 ± 21.5	81.7 ± 19.7	18	-	2
	(15.7–21.7)	(13.2–18.8)	(45.8–124.1)	(43.1–111.4)			
*Upeneus moluccensis* (PR)	17.9 ± 1.4	14.7 ± 1.2	81.4 ± 15.8	76.2 ± 16.6	15	4	1
	(15.6–20.1)	(13.0–16.6)	(57.0–114.30)	(50.8–117.2)			

^a^SL = TL

*TL*, total length; *SL*, standard length; *TW*, total weight; *SW*, slaughter weight; $\underline{x}$ ± *SD*, mean ± standard deviation; *m*, male; *f*, female; *n*.*I*., not identified. *n* = 20

### Parasitological examination

The body cavity of the fish was opened, and intestinal organs and the fillets were removed and checked for metazoan endoparasites following standard protocols by Palm (2004) and Palm and Bray (2014). Cleaned from host tissue, parasites were collected in 70% ethanol and later preserved in glycerin following Riemann (1988) and fixed on microscopic slides. Parasites identification was done according to keys and to original descriptions in the current literature (Malakhov 1986; Khalil et al. 1994; Gibson et al. 2002; Jones et al. 2005; Bray et al. 2008). After taxonomic and molecular identification, the endoparasite specimens and specimen vouchers (molecular analyses) were deposited at LIPI, Science Centre, Jakarta-Bogor, Indonesia (Accession numbers: CR1-9, 157–172, 540–541, 1436–1481). The calculation of parasitological terms such as prevalence (P), intensity (I), mean intensity (mI), mean abundance (mA) followed Bush et al. (1997).

### Stomach content analysis

The stomach content of the investigated fish was analyzed for ingested prey items. Prey identifiable to class or order level was counted and documented. The weights of the full stomach and the empty stomach and the weights of the individual prey items were taken (to 0.01 g). Percentage of frequency of occurrence (F%), numerical percentage of prey items (N%), and weight percentage of prey items (W%), as well as the index of relative importance (IRI) of each prey item, was calculated after Hyslop (1980). The four feeding categories were established based on the weight percentage of prey items in the stomachs: > 60%W teleostei = mainly fish feeder, > 60%W cephalopods = mainly cephalopod feeder, 50% teleostei, and 50% decapod = fish and decapod feeder. Furthermore, the fish feeders were divided into two groups based on their size (< 50 cm: smaller schooling fish, > 50 cm: top predator).

### Molecular analyses

The molecular analyses focused only on digenean endoparasites due to primer availability at the time of the study. Hence, three specimens of *Stephanostomum* cf. *uku* (of Bray et al. 2005) from *Alecta indica* and *Lecithochirium* sp. from *Trichiurus lepturus* were used respectively for molecular analyses and further species identification. Molecular vouchers were prepared for sequenced digeneans prior to analysis and deposited in the Indonesian Biodiversity Collection at LIPI, Cibinong, Bogor, Indonesia. Genomic DNA was extracted by using the Blood & Tissue Kit by QIAGEN. The rDNA target regions were amplified with digenean specific primers: forward WormA (5′-GCG-AAT-GGC-TCA-TTA-AAT-CAG-3′) and reverse WormB (5′-CTT-GTT-ACG-ACT-TTT-ACT-TCC-3′) for the 18S rDNA region (Littlewood and Olson 2001) and forward Zx-1 (5′-ACC-CGC-TGA-ATT-TAA-GCA-TAT-3′) and reverse 1500R (5′–GCT-ATC-CTG-AGG-GAA-ACT-TCG-3′) for the 28S rDNA region (Olson et al. 2003). PCR reactions included 5 µl extracted DNA, 25 µl Master-Mix (QIAGEN), 15 µl pure water, 2.5 µl forward, and 2.5 µl reverse primer. PCR reactions were performed in a thermocycler (GeneTouch, BIOER®) with following settings: initial denaturation at 94℃ (30 s); 40 cycles of 94℃ for 30 s, 56℃ for 30 s, and 72℃ for 2 min, and then followed by final extension for 7 min at 72℃ (Olson et al. 2003). PCR products were visualized on a 0.85% agarose gel. A 1 kb ladder was used to estimate PCR product size. PCR products were purified using the PCR Purification Kit by QIAGEN before being sequenced by Seqlab, Göttingen, Germany. The resulting 28S sequences of one digenean specimen were edited using the sequence alignment editor BioEdit^©^ (version 7.0.5.3.) identified by BLASTN and aligned with homologous sequences of *Stephanostomum* cf. *uku* from Bray et al. (2005) (see accession number DQ248219.1 (GenBank, NCBI)). The obtained sequence was deposited in Genbank under the accession number MW115577.

### Statistical analyses

All parasitological and stomach content data were processed in Microsoft Excel (version 14.0), and statistical analyses were performed using Stata (Release 15.1, StataCorp 2017). To compare whether the endoparasites community differed among the four feeding categories, a two-way ANOVA with diet (four levels) and parasite groups (three levels) as factors was performed. The model assumptions were checked by residual diagnostics. Post-hoc pairwise comparisons (Bonferroni adjusted) were conducted to infer differences between the four feeding categories.

## Results

### Stomach contents

**Fig. 2:**
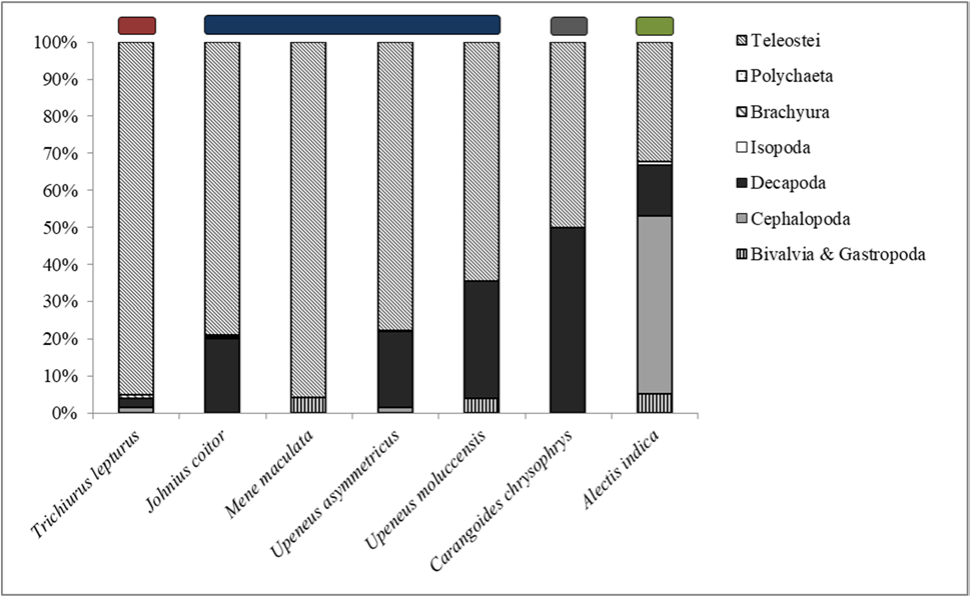
Proportion of diet items (IRI in percent) of each examined fish species. Bars indicate the four feeding categories: red: mainly fish feeder, as top predator; blue: mainly fish feeder, as smaller sized or schooling fish; gray: mainly fish and decapod feeder; and green: mainly cephalopod feeder

The diets of the seven investigated fish species consisted of seven prey items (Table 2), namely bivalves and gastropods (Mollusca), cephalopods (Mollusca), decapods (Crustacea), isopods (Crustacea), brachyurans (Crustacea), polychaetes, and bony fishes (Teleostei). Bony fishes were the most frequent diet component and occurred in all examined fish species. The second-most-frequent prey items were decapods that were recorded in six fish species. The least-frequent prey items were brachyurans, only recorded in *Trichiurus lepturus* (Table 2). Only in two fish species teleosts were not the most important diet component. *Alectis indica* fed mainly on cephalopods, and *Carangoides chrysophrys* fed in equal parts on bony fishes and decapods (Fig. 2).

**Table 2:** Percentage of frequency of occurrence (F%), numerical percentage of prey items (N%), weight percentage of prey items (W%) all in percent, and index of relative importance (IRI) of the identified food items found in examined fish species

			Stomach content						
Diet	**Fish species**		Bivalvia and gastropoda	Cephalopoda	Decapoda	Isopoda	Brachyura	Polychaeta	Teleostei
1	*Trichiurus lepturus*	F	-	15.4	23.1	-	15.4	-	92.3
		N	-	9.5	14.3	-	9.5	-	66.7
		W	-	6.0	1.8	-	0.8	-	91.5
		IRI	-	238.2	370.2	-	158.5	-	14,601.0
2	*Johnius coitor*	F	-	-	52.6	10.5	-	10.5	84.2
		N	-	-	37.8	5.4	-	5.4	51.4
		W	-	-	16.4	1.0	-	0.4	82.3
		IRI	-	-	2852.2	67.5	-	60.9	11,252.2
	*Mene maculata*	F	14.3	-	-	-	-	-	100.0
		N	10.0	-	-	-	-	-	90.0
		W	36.9	-	-	-	-	-	63.1
		IRI	670.7	-	-	-	-	-	15,305.4
	*Upeneus asymmetricus*	F	-	5.6	38.9	5.6	-	-	66.7
		N	-	3.6	28.6	3.6	-	-	64.3
		W	-	23.9	23.5	1.8	-	-	50.8
		IRI	-	152.6	2024.0	30.1	-	-	7672.0
	*Upeneus moluccensis*	F	18.2	-	54.5	-	-	-	63.6
		N	12.5	-	37.5	-	-	-	50.0
		W	11.3	-	26.4	-	-	-	62.2
		IRI	433.3	-	3487.4	-	-	-	7142.3
3	*Carangoides chrysophrys*	F	-	-	50.0	-	-	-	50.0
		N	-	-	50.0	-	-	-	50.0
		W	-	-	50.0	-	-	-	50.0
		IRI	-	-	5000.0	-	-	-	5000.0
4	*Alectis indica*	F	10.0	10.0	10.0	-	-	5.0	15.0
		N	10.5	10.5	26.3	-	-	5.3	47.4
		W	1.1	95.2	3.6	-	-	0.1	0.1
		IRI	116.2	1057.7	299.1	-	-	26.4	711.2

Fish species are listed according to their diet: (1) mainly fish feeder, as top predator, (2) mainly fish feeder, as smaller sized or schooling fish, (3) mainly fish and decapod feeder, and (4) mainly cephalopod feeder

The seven fish species were categorized into four different groups depending on the importance of the respective prey items and their position in the marine food web: (1) mainly fish feeder, as top predator (*T. lepturus*), (2) mainly fish feeder, as smaller sized or schooling fish (*Johnius coitor, Mene maculata, Upeneus asymmetricus, U. moluccensis*), (3) mainly fish and decapod feeder (*C. chrysophrys*), and (4) mainly cephalopod feeder (*A. indica*) (Fig. 2).

### Parasite fauna

We isolated a total of 11 endoparasite species from three parasite taxa (Digenea, Cestoda, Nematoda) (Table 3). *Carangoides chrysophrys* and *Upeneus moluccensis* harbored five parasite species, and *Alectis indica*, *Johnius borneensis*, *Mene maculata*, *Trichiurus lepturus*, and *U. asymmetricus* harbored four species, respectively.

**Table 3:** Overview of all found parasite taxa and their micro-and macro habitats (site and host) including prevalence (P) in percent, intensities (I), mean intensities (mI), and mean abundances (mA)

		*Trichurus lepturus*	*Johnius borneensis*	*Mene maculata*	*Upeneus asymmetricus*	*Upeneus moluccensis*	*Caranagoides chrysophrys*	*Alectis indica*				
	Diet	1	2				3	4				
Parasite species	Site								P (%)	mI	(I)	mA
Digenea												
*Lecithochirium* sp.	S			**X**					5.0	1.0	(1)	0.1
	GC, Gi, S	X							95.0	8.1	(1–33)	7.7
*Prosorhynchus* sp.	L							**X**	5.0	1.0	(1)	0.1
	S				**X**				5.0	2.0	(2)	0.1
	Int, S					**X**			10.0	1.0	(1)	0.1
*Stephanostomum cf. uku* (of Bray et al. 2005)	Int							X	35.0	2.9	(1–8)	1.0
Hemiuridae indet	Int				**X**				5.0	1.0	(1)	0.1
Cestoda												
*Callitetrarhynchus gracilis*	BC, G, Int, L, S, SB						**X**		40.0	3.3	(1–6)	1.3
	BC		**X**						5.0	1.0	(1–2)	0.1
	BC, G			**X**					10.0	2.0	(1–3)	0.2
	BC, G, GC, H, L, S	X							60.0	3.5	(1–7)	2.1
Nematoda												
*Anisakis typica* s.l.	G, Ms, P, S						X		35.0	1.4	(1–2)	0.5
	BC, G, Int		**X**						15.0	1.7	(1–3)	0.3
	L			**X**					40.0	3.0	(1–15)	1.2
	BC, G, H, Int, L, P, S	X							90.0	27.6	(1–266)	24.9
	BC				**X**				70.0	3.4	(1–10)	2.4
	BC, Int, S					**X**			80.0	2.9	(1–7)	2.4
*Camallanus carangis*	Int							**X**	15.0	1.0	(1)	0.2
	BC, Int, P		**X**						55.0	1.7	(1–4)	1.0
	P			**X**					10.0	1.5	(2)	0.2
	BC, GC, Int, L, P, S	**X**							75.0	4.5	(1–10)	3.4
	Int					**X**			5.0	1.0	(1)	0.1
*Cucullanus bulbosus*	BC, Int, P, S						X		15.0	1.7	(1–3)	0.3
*Hysterothylacium* sp.	G, H, Int, L, Ms, P, S							**X**	70.0	19.1	(1–93)	13.4
	BC, G, H, Int, L, P, S						**X**		95.0	4.1	(1–9)	3.9
	G, Int		**X**						10.0	1.0	(1)	0.1
	Int				**X**				100.0	15.1	(1–77)	15.1
	BC, Int, P, S					X			50.0	2.4	(1–7)	1.2
Nematoda indet. 1	G, Int, L, Ms, P, S						X		100.0	114.7	(31–236)	114.7
Nematoda indet. 2	BC, Int, P					X			25.0	1.0	(1)	0.3

*BC*, body cavity; *F*, fin; *G*, gonads; *GC*, gill cavity; *Gi*, gills; *H*, heart; *Int*, intestine; *L*, liver; *Ms*, mesentery; *O*, operculum; *P*, pyloric caeca; *S*, stomach; *SB*, swim bladder. Fish species are listed according to their diet: (1) mainly fish feeder, as top predator, (2) mainly fish feeder, as smaller sized or schooling fish, (3) mainly fish and decapod feeder, and (4) mainly cephalopod feeder. X = no new host or locality record, **X** = new host record, $\underline{\mathrm X}$ = new locality record, and **X** = new host and locality record

**Fig. 3:**
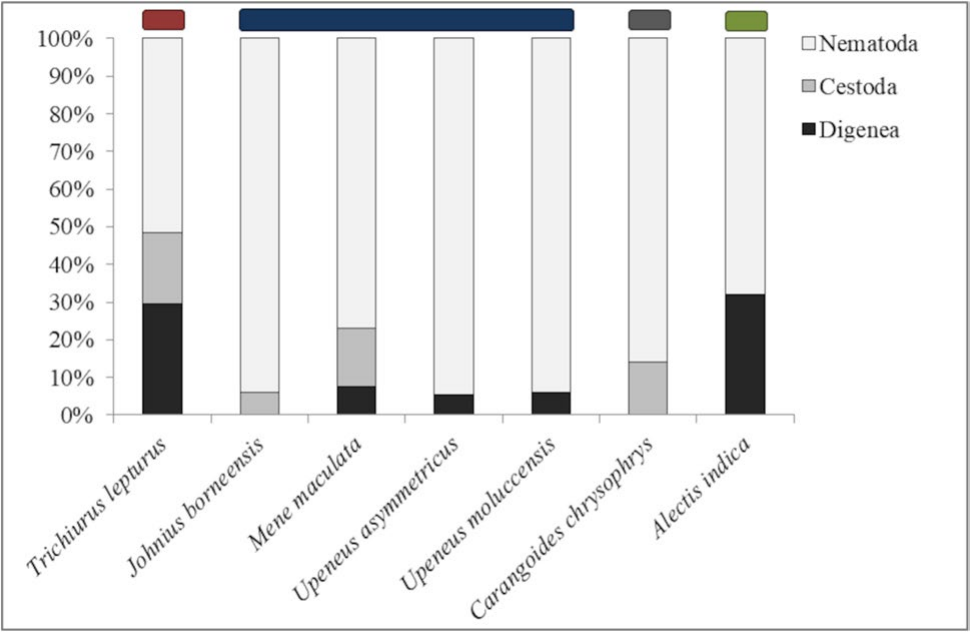
Proportion (based on prevalence data, given in percent) of parasite taxa in each examined fish species. Bars indicate the four feeding categories: red: mainly fish feeder, as top predator; blue: mainly fish feeder, as smaller sized or schooling fish; gray: mainly fish and decapod feeder; and green: mainly cephalopod feeder

Nematodes were the parasite group with the highest number of species and also the most prevalent group as all examined fishes hosted at least two nematode species. The most abundant nematode was *Anisakis typica* s.l. in *T.* *lepturus* (mA = 24.9), *Hysterothylacium* sp. in *Upeneus moluccensis* (mA = 15.1), and Nematoda indet. 1 in *Carangoides chrysophrys* (mA = 114.7). Nematodes infected nearly all inner organs (body cavity, gonads, heart, intestine, liver, mesentery, pyloric caeca, stomach, swim bladder) as well as the gill cavity, but no nematodes were found in the fillet (Table 3). The second-largest parasite group were digeneans, with six documented species. Only two fish species (*C.* *chrysophrys* and *Johnius borneensis*) harbored no digeneans, and *Mene maculata* harbored only one species. The trypanorhynch *Callitetrarhynchus gracilis* was the only documented cestode and infected four different hosts (*C. chrysophrys, J. borneensis, M. maculata*, and *T. lepturus*) in the final larval (plerocercus) stage (Fig. 3).

The data supported a significant association between the four feeding categories and the endoparasite composition (ANOVA, *F* = 194.59, *p* = 0.000, Table 4). The pairwise comparisons (Bonferroni adjusted) showed that *Trichiurus lepturus* (feeding category one, predatory fish feeder) had a different endoparasite composition compared to fishes in the other three feeding categories (*p* = 0.000). *T. lepturus* had the evenest distribution of parasite groups with 52% nematodes, 30% digeneans, and 19% cestodes, while smaller-sized fish feeders (feeding category harbored a higher proportion of nematodes (> 70%). *Carangoides chrysophrys* (feeding category three, fish and decapod feeder) and *Alectis indica* (feeding category four, cephalopod feeder) also had a significantly different endoparasite composition (*p* = 0.007). *C.* *chrysophrys* was highly infected with nematodes (86%) but hosted no digeneans but cestodes, while *A.* *indica* harbored the highest proportion of digeneans (32%) and no cestodes. The endoparasite composition in smaller-sized fish feeders (feeding category two) did not differ from *C. chrysophrys* or *A. indica*.

**Table 4:** Results of analysis of variance (ANOVA) comparing parasite groups by feeding category (diet)

Source	DF	Sum of squares	Mean of squares	*F*-test	*p*-value
Diet	3	169.30	56.43	194.5	0.0000
Residual	3961	1148.79	0.29		

*DF*, degrees of freedom

### Molecular analysis

Both the 18S DNA region and the 28S DNA region of six isolated digeneans were processed within the present study, and *Stephanostomum* cf. *uku* (of Bray et al. 2005) was identified through molecular analysis for the first time from Indonesian waters. The sequence from the 28S region of *Stephanostomum* sp. (by morphological determination) from *A. indica* (see accession number MW115577) matched a reference sequence of *Stephanostomum* cf. *uku* from *Aprion virescens* in Lizard Island, Australia, reported by Bray et al. (2005) (see accession number DQ248219.1) in Genbank NCBI via BlastN 2.8.1 (100% percent identity). For the remainder of the manuscript, we will refer to the here-identified species as *Stephanostomum* cf. *uku* (of Bray et al. 2005).

## Discussion

The present study was the first parasitological investigation of *U.* *asymmetricus* worldwide and of *C. chrysophrys* in Indonesia. In total, the fishes hosted 11 endoparasite species and fed on seven different prey items. We established 22 new host and 14 new locality records and extended the knowledge of endoparasites in Indonesia by 24% in *A. indica*, 25% in *C. chrysophrys*, 40% in *J. borneensis*, 44% in *M. maculata*, 100% in *U.* *asymmetricus*, and 17% in *U. moluccensis*.

### Endoparasite fauna

We found that nematodes were the most predominant parasite group. They were represented with six taxa, infected hosts with the highest prevalence (up to 100%), and showed the highest intensities (Table 3).

Due to their complex life cycles and many intermediate hosts, nematodes infect a wide range of phyla (Køie 2001; Shamsi 2014) and their zoonotic potential has been discussed in recent years in Indonesia (Palm et al. 2008; Klimpel and Palm 2011; Dewi and Palm 2017). We isolated two nematode taxa of potentially zoonotic genera: *Anisakis typica* s.l. and *Hysterothylacium* sp. (Ishikura 1989; Shamsi et al. 2018). The here-reported *A. typica* s.l. is likely identical to *A. typica* var. *indonesiensis* which is the predominant genotype in Indonesia (Palm et al. 2017). Larval stages of *Anisakis* spp. can cause anisakiasis and allergic reactions in humans when ingested along with raw or undercooked fish (Sakanari and McKerrow 1989; Audicana and Kennedy 2008; Aibinu et al. 2019). A recent review on anisakiasis by Aibinu et al. (2019) suggested its worldwide occurrence, and a seroepidemiological survey done on a Javanese population reported *Anisakis* antibodies in 11% of the 244 study subjects. This indicates that anisakid-borne zoonoses are a risk in Indonesia. Although *A. typica* has not been associated with human infections and this species mainly infects the gastrointestinal tract of the host, post mortem migration of helminth larvae into the fillet – from which transmission to humans can take place – has been recorded in anisakid worms and is a potential threat to consumer health (Bao et al. 2017; Shamsi et al. 2018). With regards to the zoonotic potential, similar patterns have also been recorded for *Hysterothylacium* spp., where larvae can cause disease in consumers after originally infecting the intestine but subsequently migrating to the fillet (Shamsi et al. 2018). Therefore, zoonoses could still be a health risk to fish consumers in Indonesia, requiring more thorough investigation.

Each fish species hosted digeneans except for *C. chrysophrys* and *J.* *borneensis* (Table 3). With four different species, digeneans were the second-most diverse parasite group but were found with a lower prevalence than Nematoda. However, when we compare our study to previous parasitological investigations in Indonesia, there is no noticeable decrease in digenean abundance (e.g., Palm and Rueckert 2009; Rueckert et al. 2009a; Kleinertz et al. 2014). *Stephanostomum uku* has been previously reported only from *Aprion virescens* (family: Lutjanidae), a large benthopelagic predator (Froese and Pauly 2021) in Hawaii (Yamaguti 1970), New Caledonia (Bray and Justine 2011), and Australia (Bray et al. 2005), the latter providing the reference sequence used in this study. Finding *Stephanostomum* cf. *uku* (of Bray et al. 2005) for the first time in Indonesia from a carangid fish highlights the importance of using molecular techniques to better understand the complex and diverse parasite fauna in tropical regions, especially under consideration of the potentially wide range of distributions.

It was suggested by Palm (2004) that Indonesia is a hotspot for the cestode order Trypanorhyncha. In Pelabuhan Ratu, more than 50 trypanorhynch species were recorded (Palm 2004; Haseli et al. 2010), and Jakob and Palm (2006) reported five species from *Trichiurus lepturus* alone, including *Callitetrarhynchus gracilis*. Here, only one species, *C. gracilis*, was identified. Trypanorhynch abundance relates to the relative abundance and species richness of their elasmobranch final host, meaning that if the number of final hosts decreases so will the parasite (Haseli et al. 2010). Indonesia is the world’s largest shark-fishing nation (Dent and Clarke 2015), and elasmobranchs do not recover easily from overfishing (Myers et al. 2007), so the low-observed abundance and diversity of cestodes could indicate a low elasmobranch abundance in the region. Tetraphyllideans (order: Cestoda) commonly infect *Trichiurus lepturus* (Jakob and Palm 2006; Theisen 2020) and larger-predatory ocean fishes. A reduction in the elasmobranch population as the final hosts may also explain the absence of tetraphyllideans in the present study.

(Rueckert et al. 2009a) isolated 14 endoparasite species from *Upeneus moluccensis* (*n* = 30) in Lampung Bay, southern Sumatra, Indonesia. A higher number of parasite species might correlate with a higher number of analyzed fishes, but it also indicates variability in parasite distribution. Another study from the same year in Segara Anakan, southern Java, Indonesia, isolated five endoparasites from *Johnius coitor* (*n* = 20), a congener of *Johnius borneensis* and one endoparasites species (*Anisakis* sp.) from the carangid *Caranx sexfasciatus* (*n* = 8) (Rueckert et al. 2009b). In terms of parasite richness, these results are more similar to what was observed in the present study. For conclusive results on potential decreases in endoparasites richness and shifts in their host’s distribution, consistent parasitological surveys are necessary, which would benefit the monitoring of intermediate and final host species as well as enhance seafood consumer safety with regards to zoonotic species.

With numerous new host and new locality records, we show the large potential of endoparasite research in highly diverse Indonesian waters. Finding endoparasites which are a potential risk to seafood consumers and can also negatively impact fish health in every fish species (*A*. *typica s.l.*, *Hysterothylacium* sp.) stresses the importance of closing knowledge gaps in the marine parasite fauna. Many here-isolated endoparasites infect a wide range of fish hosts and can be transmitted along with the food web (Rigaud et al. 2010) which poses transmission risks into other wild and mariculture fish species. Parasitological research on food fishes in Indonesia is essential to assess food safety to consumers and to predict and prevent disease outbreaks.

We would like to address that, although the standardized parasitological protocols used here should describe the endoparasite community as best as possible (Palm 2011), no overnight incubation of the gastrointestinal tract has been done because frozen fish were used for which this method does not apply (Shamsi and Suthar 2016). Incubation is especially useful for nematode detection, so it can be assumed that parasitological parameters (mean intensity, intensity, mean abundance) for this group may be higher than reported in this study. This means that the risk of parasite-borne zoonoses in food fish in Indonesia may be even higher than reported here.

### Feeding ecology

The feeding categories in which the seven fish species were categorized based on the observed stomach contents that mainly corresponded to the previously recorded diet according to the literature. *Trichiurus lepturus* (feeding category one) is a larger predator feeding mainly on fish and squid (Nakamura and Parin 1993; Abidin et al. 2013), and here, the diet consisted nearly exclusively of teleosts. The diet of *Mene maculata* and *Upeneus asymmetricus* has never been analyzed before, and here, we provide the first dietary data for these species. *Johnius borneensis* feeds mainly on fishes and crustaceans, which is compliant with the present study (Theisen 2009). As bottom dwellers, members of the genus *Upeneus* mainly feed on benthic invertebrates, fish, and polychaetes (Kaya et al. 1999; Campos-Dávila et al. 2002; Prabha and Manjulatha 2008). Here, in both *U. asymmetricus* and *U. moluccensis*, fish dominated the diet and crustaceans only played a minor role. *Carangoides chrysophrys* (feeding category three) mainly feeds on teleosts and crustaceans, similar to what was observed here (Al Kamel and Kara 2019). *Alectis indica* (feeding category four) is reportedly a piscivorous species feeding also on squids and crustaceans (De Troch et al. 1998; Froese and Pauly 2021), which is similar to our findings.

Diet and the accumulation of parasites are closely linked in aquatic ecosystems (Marcogliese 2004, 2005). Therefore, it was of interest whether the isolated parasites can be attributed to the feeding ecology of the host. We showed that the endoparasite composition differs according to the four different feeding categories. This coincides with our hypotheses that based on their ecology and diet, fishes are likely to accumulate certain endoparasites. Interestingly, the endoparasite composition of *T.* *lepturus* could be distinguished from fishes in the other feeding categories as it had the most evenly distributed parasite fauna, with all three parasite groups accounting for at least 19%. As a top predator, *T. lepturus* may be able to feed on a larger range of teleosts which in turn leads to a more diverse accumulation of endoparasites (Lile 1998; Yunrong et al. 2011). Smaller-sized piscivores (feeding category 2) seem to accumulate many nematodes, potentially because these parasites are transmitted into their food source (small fish) via a large range of intermediate hosts such as shrimp, isopods, and amphipods (Klimpel and Rueckert 2005). Crustaceans are an important food source for fishes and are known to be intermediate hosts for a wide range of marine endoparasites (Klimpel and Rueckert 2005; Koehler and Poulin 2010; Busch et al. 2012). Here, *C. chrysophrys* (feeding category three) had a high nematode prevalence, potentially associated with higher consumption of decapods. Squid have been found to be paratenic hosts of especially anisakid worms and cestodes (Petrić et al. 2011), and although *Alectis indica* (feeding category four) hosted mainly nematodes, no cestodes were isolated from this species.

Although this study had a limited sample size and therefore limited statistical power, we show that an association between the feeding ecology and diet in food fishes in Indonesia is possible. While stomach content analysis provides information about a short time span, parasite data reflects the fish ecology in a longer term: Food items remain in the fish stomach until digested while some long-lived parasites can remain in their hosts for several years (Marcogliese 2003; Palm and Rueckert 2009; Palm 2011). While more information about the diet can provide important insights about predator–prey relationships (Al Kamel and Kara 2019), a combined approach of parasitological studies and stomach content analysis can be an efficient tool to fill knowledge gaps in Indonesian fish ecology and to work toward better ecosystem-based management plans. Climate change and anthropogenic activities have and likely will in the future impact marine biodiversity (Asch et al. 2018; Worm and Lotze 2021). Changes in marine food webs and zoogeographical distributions of intermediate and final hosts go along with changes in parasite abundance and infection patterns (Shamsi 2021). This could introduce marine parasitic zoonoses or parasitic fish diseases to new regions and call for more parasitological research in affected regions.

## Data Availability

If wanted, we can share our raw data.
